# Effect of Tomato Industrial Processing on Phenolic Profile and Antiplatelet Activity

**DOI:** 10.3390/molecules180911526

**Published:** 2013-09-17

**Authors:** Eduardo Fuentes, Oscar Forero-Doria, Gilda Carrasco, Adolfo Maricán, Leonardo S. Santos, Marcelo Alarcón, Iván Palomo

**Affiliations:** 1 Department of Clinical Biochemistry and Immunohematology, Faculty of Health Sciences, Interdisciplinary Excellence Research Program on Healthy Aging (PIEI-ES), University of Talca, Talca 3460000, Chile; E-Mails: edfuentes@utalca.cl (E.F.); malarcon@utalca.cl (M.A.); 2 Centro de Estudios en Alimentos Procesados (CEAP), CONICYT-Regional, Gore Maule, R09I2001, Talca, Chile; 3 Laboratory of Asymmetric Synthesis, Institute of Chemistry and Natural Resources, University of Talca, Talca 3460000, Chile; E-Mails: oforero@utalca.cl (O.F.-D.); amarican@utalca.cl; lssantos@utalca.cl (A.M.); 4 Horticulture Department, Faculty of Agricultural Sciences, University of Talca, Talca 3460000, Chile; E-Mail: gcarrasc@utalca.cl

**Keywords:** tomato, tomato products, antiplatelet activity, phenolic compounds, nutrition

## Abstract

*Background*: Regular consumption of fruits and vegetables (e.g., tomatoes) has been shown to be beneficial in terms of reducing the incidence of cardiovascular diseases. The industrial processing of tomatoes into tomato-based products includes several thermal treatments. Very little is known on the effect of tomato industrial processing on antiaggregatory activity and phenolic profile. *Methods*: It was assessed the effect of tomato and by-products extracts on platelet aggregation induced by ADP, collagen, TRAP-6 and arachidonic acid. These *in vitro* antithrombotic properties were further supported in an *in vivo* model of thrombosis. A set of antiplatelet compounds has been selected for HPLC analysis in the different extracts. *Results*: Some natural compounds such as chlorogenic, caffeic, ferulic and *p*-coumaric acids were identified by HPLC in tomatoes and its products may inhibit platelet activation. Red tomatoes, tomato products (sauce, ketchup and juice) and by-products extracts inhibited platelet aggregation induced adenosine 5'-diphosphate, collagen, thrombin receptor activator peptide-6 and arachidonic acid, but to a different extent. Also, pomace extract presents antithrombotic activity. *Conclusions*: Processed tomatoes may have a higher content of health-benefiting compounds than fresh ones. Pomace even presents the best antiplatelet activity. Finally, tomato products may be used as a functional ingredient adding antiplatelet activities to processed foods.

## 1. Introduction

The prevalence of cardiovascular diseases (CVD) (*i.e.*, acute myocardial infarction, cerebrovascular disease and peripheral arterial thrombosis) has increased significantly in recent years [1,2]. The development and progression of CVD lies in the interactive processes of atherosclerotic lesions and thrombus formation, an interaction established primarily by platelet participation [3]. Platelets play an important in CVD, even platelet hyper-aggregability is also associated with the risk factors for CVD (e.g., smoking, hypertension and hypercholesterolaemia) [4]. In the context of atherosclerosis, platelets can adhere to endothelial cells and contribute to the recruitment of leukocytes involved in the local vascular inflammation and thrombosis formation [5,6]. In this sense, the inhibition of the platelet function has been used for a long time in an effort to prevent and treat CVD [7].

There is increasing evidence that high consumption of fruit and vegetables (F&V) is beneficial for CVD prevention [8]. The well-known correlation between diet and health demonstrates the great possibilities of food to maintain or even improve health. This fact has attracted a great interest in searching for new products that contribute to improving our health and well-being [9]. Furthermore, the high consumption of tomatoes provide antiplatelet activity [10]. In addition, it was recently isolated and identified adenosine and guanosine from tomato [11,12]*.* Guanosine and adenosine showed a potent antiplatelet activity through the inhibition of platelet secretion, adhesion and aggregation [11,12]. 

According to their micronutrient content, tomatoes, and therefore tomato-based products, also contain valuable phytochemicals or bioactive components: mainly phenolic compounds and carotenoids, such as lycopene. Phenolic compounds in tomatoes are mainly represented by flavanones (naringenin glycosilated derivatives) and flavonols (quercetin, rutin and kaempferol glycosilated derivatives) [13]. The industrial processing of tomatoes into tomato-based products includes several thermal treatment steps such as drying, heating and pasteurizing. 

Recently, aqueous and methanolic extracts of red tomato were found to be thermally stable in the temperature range of 20 to 100 °C. Also both, acid and alkali were not affected inhibition of platelet aggregation induced by ADP [10]. Industrial processing of tomato generates a considerable amount of waste, peel, seeds and a part of the pulp, which are known as tomato pomace [14]. A classic method for industrial processing of tomatoes into tomato-based products includes several thermal treatment steps such as drying, heating and pasteurizing [15]. During this processing, several additional changes can occur to affect the appearance, composition, nutritional value and sensory properties (color, texture and flavor of the product) [16]. Even processed tomatoes may have a lower content of health-benefiting compounds than fresh ones [17]. In this sense, little is known about the effects of these treatments on antiplatelet activity and its relation to the polyphenol content. The main aim of this work was to investigate the antiplatelet activity of aqueous extract from red tomatoes, tomatoes products (sauce, ketchup and juice) and pomace. Moreover, it was identified and quantified phenolic compounds that provide this activity.

## 2. Results and Discussion

### 2.1. Chromatographic Analysis of Extracts

HPLC analysis of aqueous extracts from red tomatoes, tomatoes products (sauce, ketchup and juice) and pomace revealed a group of phenolic compounds, which have been identified as chlorogenic, ferulic, *p*-coumaric and caffeic acids (Table 1). 

**Table 1:** Quantification of phenolic compounds in extracts from red tomatoes, tomatoes products (sauce, ketchup and juice) and pomace.

Extracts	Phenolic compounds (mg/kg)			
	Chlorogenic acid	Caffeic acid	*p*-Coumaric acid	Ferulic acid
*Tomatoes*	ND	6,765.7 ± 4 ^a^	10,209.8 ± 3 ^a^	4,786.7 ± 3 ^a^
*Sauce*	101.5 ± 2 ^a^	ND	7.9 ± 1 ^b^	ND
*Ketchup*	181.3 ± 2 ^b^	55.6 ± 2 ^b^	8.9 ± 1 ^b^	7.9 ± 1 ^b^
*Juice*	233.8 ± 3 ^c^	55.2 ± 1 ^b^	25.1 ± 2 ^c^	25.9 ± 2 ^c^
*Pomace*	75.6 ± 1 ^a^	51.2 ± 2 ^b^	24.9 ± 2 ^c^	2.3 ± 1 ^b^

^a, b, c^ Values are presented as mean ± S.E.M (n = 3) that with different letters are significantly different at *p* < 0.05. ND: not detected.

In aqueous extracts from sauce, ketchup, juice and pomace the total amount of caffeic, *p*-coumaric and ferulic acids was markedly decreased during industrial tomato processing. Thus, based on HPLC determination, the content of phenolic compounds in aqueous extract from red tomatoes is in the following order: ferulic (4,786.71 ± 1 mg/kg), caffeic (6,765.73 ± 2 mg/kg) and *p*-coumaric acids (10,209.79 ± 2 mg/kg). Similar compounds have been reported by ^1^H-NMR using tomato extracts [13].

### 2.2. Antiplatelet Activity of Bioactive Compounds

The design of new therapeutic strategies targeting platelets, and their impact in the atherosclerotic-related inflammatory response and the subsequent thrombotic event are in the center of current cardiovascular research [18,19]. Interestingly, some natural compounds consumed regularly in the diet may inhibit platelet activation pathways. More specifically, a number of dietary components including some fats, nucleosides and among others have shown to diminish platelet activation [11,20]. The effects of phenolic compounds (chlorogenic acid, caffeic acid, ferulic acid and *p*-coumaric acid) on ADP-, collagen-, TRAP-6- and AA- induced platelet aggregations are presented in Table 2. Chlorogenic and caffeic acids effectively reduced ADP-, collagen-, TRAP-6- and AA-induced platelet aggregation (*p* < 0.05). Moreover, *p*-coumaric acid suppressed ADP-, collagen-, TRAP-6-induced platelet aggregation (*p* < 0.05). Ferulic acid only inhibited platelet aggregation induced by ADP and collagen (*p* < 0.05). In this sense, epidemiological studies have provided evidence of a protective role of healthy diets in the prevention of CVD [21]. To this respect, our group has recently isolated and identified adenosine and guanosine as a bioactive compound in *Solanum lycopersicum* (a cherry type tomato) with potent antiplatelet activity [11,12]. 

**Table 2:** Antiplatelet activity of phenolic compounds.

Compounds	Platelet antiaggregant activity (%)			
	ADP	Collagen	TRAP-6	AA
Chlorogenic acid	69 ± 5 *	50 ± 5 *	19 ± 4 *	22 ± 5 *
Caffeic acid	35 ± 5 *	42 ± 6 *	25 ± 5 *	20 ± 3 *
Ferulic acid	47 ± 4 *	36 ± 3 *	NS	NS
*p*-Coumaric acid	71 ± 6 *	69 ± 5 *	41 ± 6 *	NS

Values are presented as mean ± SEM (n = 3). ADP 8 µmol/L, collagen 1.5 μg/mL, TRAP-6 30 µmol/L and AA 1 mmol/L. Phenolic compounds at 0.5 mmol/L. * *p* < 0.05 *vs*. negative control (saline 0.9%). NS = no significance.

### 2.3. Antiplatelet Activity of Tomatoes and Its Derivates

**Figure 1:**
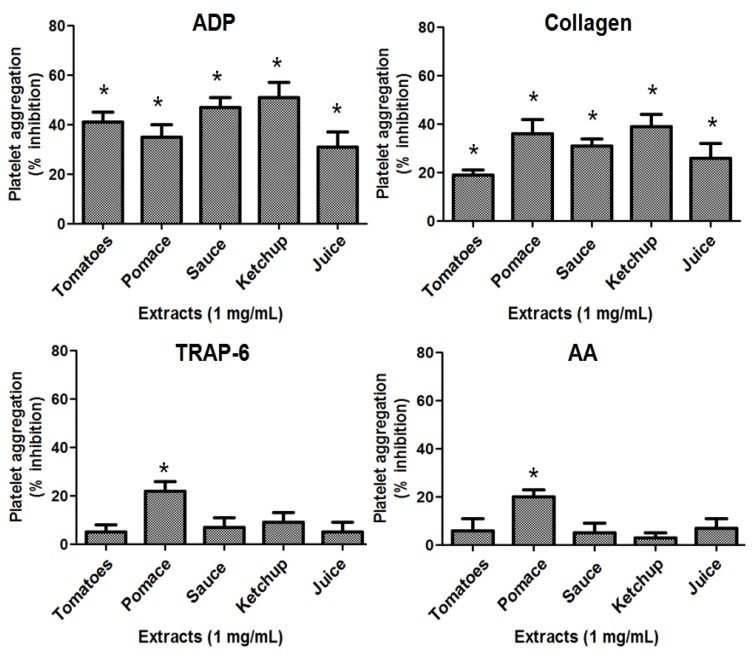
Quantitation of the inhibitory effect of tomato and its products on platelet aggregation induced by ADP (8 µmol/L), collagen (1.5 μg/mL), TRAP-6 (30 µmol/L) or AA (1 mmol/L). Results were expressed as % inhibition (mean ± SEM, n = 3). *p<0.05 *vs*. negative control (saline 0.9%).

The results of platelet aggregation induced by the agonists ADP, collagen, TRAP-6 and AA, respectively, with added extracts from red tomatoes, tomatoes products (sauce, ketchup and juice) and pomace are presented in Figure 1. Both extracts from tomatoes and its products (sauce, ketchup and juice) inhibited platelet aggregation induced by ADP and collagen, respectively, but to a different extent. Thus the inhibition of platelet aggregation induced by ADP compared to control (*p* < 0.05) was observed in the following order: ketchup > sauce > tomatoes > pomace > juice. With respect to collagen induced platelet aggregation, the inhibition compared to control (*p* < 0.05) was in the following order: ketchup > pomace > sauce > juice > tomatoes.

Tomato products and especially concentrated tomato paste are important sources of antioxidants in the Mediterranean diet. Tomato fruit contain well-known antioxidants such as vitamin C, carotenoids, flavonoids and hydroxycinnamic acids. The industrial processing of this fruit into tomato paste involves several treatments that potentially affect the final profile of antioxidants and other metabolites in the commercial product [22]. In this article, processed tomatoes may have a higher content of health-benefiting compounds. In this sense, it is possible to observe that tomato products (sauce, ketchup and juice) contain bioactive compounds that have significant antiplatelet activity (chlorogenic, caffeic, *p*-coumaric and ferulic acids).

Pomace extract presents the best antiplatelet activity [14] because it inhibits platelet aggregation by different activation pathways: ADP, collagen, TRAP-6 and AA. Pomace is a byproduct of industrial processing of tomatoes into paste and canned products. It mainly consists of seeds and the peel [23]. However, apart from lycopene pomace still contains other valuable compounds exerting complementary biological activities as antiplatelet activity (chlorogenic, caffeic, *p*-coumaric and ferulic acids). This form of pomace extract exerted a potent inhibition of platelet aggregation induced by ADP, collagen, TRAP-6 and AA. Considering the different agonists tested in this study, the inhibition of platelet aggregation by the pomace extract was in the following order: collagen (36 ± 6%) > ADP (35 ± 5%) > TRAP-6 (22 ± 4%) > AA (20 ± 3%) as compared to control (*p* < 0.05). Although the pomace is a product of industrial processing of tomatoes, it presents high amounts of crude fiber and protein [24]. Therefore, the intake of functional compounds from pomace may be associated with the reduction of cardiovascular risk through antiplatelet activity.

### 2.4. Antithrombotic Activity of Pomace Extract

**Figure 2:**
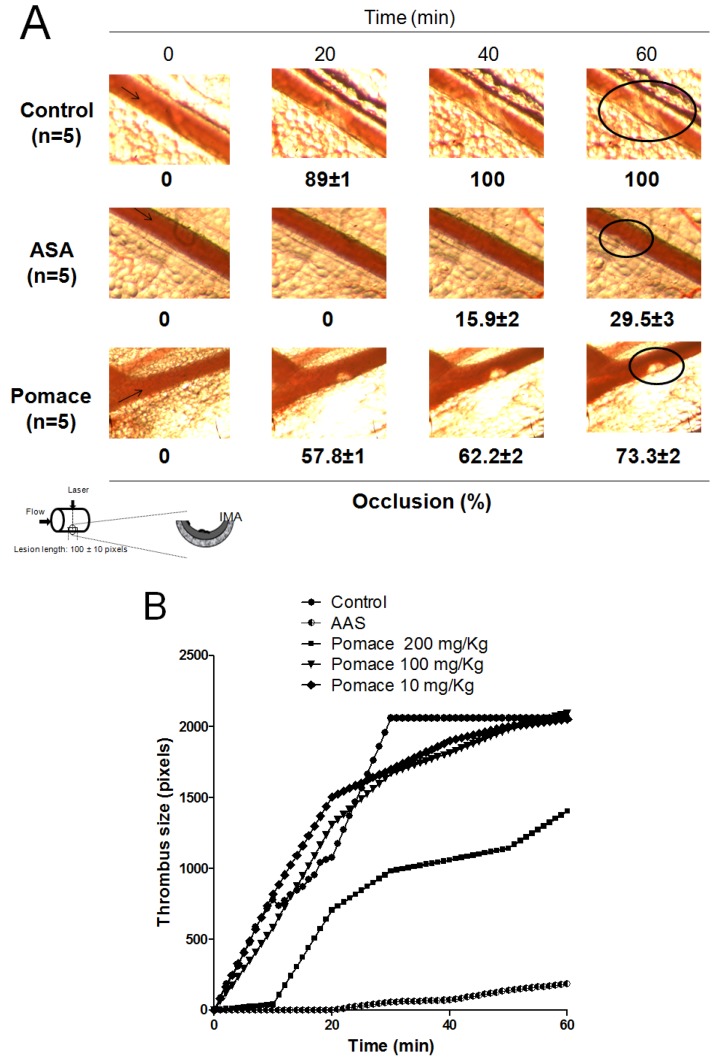
Pomace extract inhibits laser-induced thrombus formation in mesenteric artery of C57BL/6 mice. (**A**) Representative images of thrombus formation at baseline, 20, 40 and 60 min after laser-injured vascular injury in mouse mesenteric artery. (**B**) Representative time course changes of thrombus growth rate among the control (saline), pomace extract and ASA at 60 min after laser irradiation.

Antithrombotic therapy allows a reduction in deaths from heart attacks, the risk of stroke in people with heart irregularities (atrial fibrillation) and mini-strokes [25]. The dramatically inhibitory effects of pomace extract on platelet aggregation, induced by ADP, collagen, AA and TRAP-6 supported the assessment of pomace extract effectiveness to inhibit *in vivo* thrombus formation. To examine the *in vivo* antithrombotic activity of pomace extract, we evaluated the effects of pomace extract on laser-injured thrombus formation in mice mesenteric artery *in vivo*. As shown in Figure 2, in untreated mice (control), the mesenteric artery was totally blocked by a stable bulky thrombus at 20 min. In contrast, further analysis revealed that the time to form the artery thrombosis was prolonged by pomace extract-treated mice compared to the mice receiving the same volume of vehicle. Thus, one intraperitoneally bolus injection of pomace extract (200 mg/kg) 30 min before laser injury prevented thrombus formation over 60 min after laser injury, only achieving a maximum of occlusion of 73.3 ± 2% (*p* < 0.01 *vs*. control) (Figure 2).

## 3. Experimental

### 3.1. Chemicals and Reagents

Formic Acid (p.a), sodium chloride (p.a.), ethanol (p.a), methanol and acetonitrile (Lichrosolv^®^, gradient grade) were obtained from Merck (Darmstadt, Germany) and used without further purification. Chlorogenic acid, ferulic acid, *p*-coumaric acid and caffeic acid were supplied by Sigma-Aldrich (St. Louis, MO, USA). Each standard solution was prepared in methanol to obtain solutions with 1,900, 5,400, 5,600 and 6,100 ppm, respectively. Then, for each compound a calibration curve was prepared mixing these standards in ethanol 12% and milliQ water, to reach concentrations of 0.1–30 ppm. Adenosine 5'-diphosphate (ADP), thrombin receptor activator peptide 6 (TRAP-6), collagen, arachidonic acid (AA) and acetylsalicylic acid (ASA) were also obtained from Sigma-Aldrich. 

### 3.2. Processing Material

*Cluster tomatoes*. Processed tomatoes corresponding to late season hybrids (H9665, H7709 and H9997) with 135 days were obtained from “Sugal Chile” (previously “Tresmontes Luchetti”, production plant Talca, Talca, Chile). The sample collection was performed using completely random sampling from properties located in Maule Region, Chile. The tomatoes used to produce tomato products and pomace were selected from samples with the same growth and climatic conditions.

*Tomato products*. Sauce, ketchup and juice were obtained from the Regional Supply Centre of Talca, Chile.

*Pomace*. Samples composed of seeds and peels resulting from industrial tomato paste processing were obtained from “Sugal Chile” (previously “Tresmontes Luchetti”, production plant Talca, Talca, Chile). With respect to the hybrids used for pomace production, given the production line of the plant, it was not possible to define the hybrid types. Therefore, only was possible to identify the studied blends including middle growing cicle pomace cultivars (128 days: Sun6366, AB3 and HMX7883) and late (135 days: H9665, H7709 and H9997).

### 3.3. Preparation of Extracts

Aqueous extracts from red tomatoes, tomatoes products (sauce, ketchup and juice) and pomace were obtained according to Fuentes *et al*. [11]. Briefly, the samples were macerated at room temperature with water. The resulting extract was centrifuged (6,000 *g*) for 5 min and the supernatant lyophilized and stored at −20 °C until use. The lyophilized extracts were dissolved twice in ethanol 12% and milliQ water, using an ultrasonic bath, filtered using syringe filters (Millex, PVDF 0.45 μm membrane), prior the HPLC separation.

### 3.4. Instrument

The quantitative study of the phenolic compounds in all samples was performed by HPLC. The HPLC system (Agilent Technologies 1260 Infinity) consisted of a high pressure quaternary pump, a diode arrangement detector (DAD), and autosampler with 99-vial capacity sample. Separations were achieved on a LiChrocart^®^ 250-4 RP-18 (250 mm × 4 mm ID × 5 μm) (Merck). The chromatographic conditions were the following: mobile phases were prepared with formic acid 4.5% and milliQ water (solution A). These solutions were filtered through Millipore nylon filters (0.45 μm). Acetonitrile gradient grade (solution B), gradient programmed, initial 98% A/2% B, 0 to 10 min; 80% A/20% B, 10 to 15 min; 78.5% A/21.5% B, run time 15 min, injection volumes 40 μL, wavelength 280 nm. The chromatographic column worked at room temperature and flow rate were optimized for phenol separation: initial 1.2 mL/min for 10 min and then 1.3 mL/min for 10 to 15 min. Analytes in each sample were identified by comparing their retention times and UV-Vis spectra with those of standard compounds or literature data. Peak purity was checked to exclude any contribution from interfering peaks. Results were expressed as mg compound in 1 kg of the dried extract (mg/kg). Each measurement was performed in triplicate.

### 3.5. Preparation of Human Platelet Suspensions

Venous blood samples were taken from two young healthy volunteers (who had previously signed informed consent) in 3.2% citrate tubes (9:1 v/v) by phlebotomy with a vacuum tube system (Becton Dickinson Vacutainer Systems, Franklin Lakes, NJ, USA). The ethic committee of Universidad de Talca authorized the protocol in accordance with the Declaration of Helsinki (approved by the 18th World Medical Assembly in Helsinki, Finland, 1964). Tubes were centrifuged (DCS-16 Centrifugal Presvac RV) at 240 *g* for 10 min to obtain platelet-rich plasma (PRP). PRP was adjusted to 200 × 10^9^ platelets/L with platelet-poor plasma (PPP) obtained by centrifugation of the original tubes at 650 *g* (10 min). Platelet counts were performed in a hematologic counter (Bayer Advia 60 Hematology System, Tarrytown, NY, USA).

### 3.6. Platelet Aggregation Assay

Platelet aggregation was monitored by light transmission according to Born and Cross [26], using a lumi-aggregometer. Briefly, 480 μL of PRP (200 × 10^9^ platelets/L) were pre-incubated with 20 μL of saline, ASA (0.3 mmol/L) or extract/compounds (1 mg/mL or 0.5 mmol/L) for 3 min. Then, 20 μL of agonist (ADP 8 µmol/L, collagen 1.5 μg/mL, TRAP-6 30 µmol/L or AA 1 mmol/L) was added and platelet aggregation registered during 6 min. All measurements were performed in triplicate. Results of platelet aggregation (maximal amplitude [%], slope, area under the curve and lag time [s]) were determined by using the software AGGRO/LINK (Chrono-Log, Havertown, PA, USA). Inhibition of the maximal platelet aggregation was expressed as a percentage of the control (saline).

### 3.7. *In Vivo* Murine Model of Thrombosis

Thrombosis in mice was induced by photochemical injury using a modification of the methods described by Przyklenk and Whittaker [27]. Briefly, C57BL/6 mice (14–18 weeks old) were anesthetized with a combination of tribromoethanol (270 mg/kg) and xylazine (13 mg/kg). The mesentery was exposed by performing a central incision in the abdomen, permitting the visualization of thrombus development in mesenteric vessels. Thrombosis was induced by injection of 50 mg/kg Rose Bengal through the tail vein followed by illumination of the exposed mesenteric area with a 1.5-mW green light laser (532 nm). Blood flow was monitored for 60 min and stable occlusion was defined as a blood flow of 0 mL/min for 3 min. Saline (control group, n = 3), ASA (200 mg/kg, n = 3) or pomace extract (200 mg/kg, n = 3) were administered intraperitoneally 30 min before the experiment. Rectal temperatures were similar and within the physiological range among all experimental animals throughout the experimental period.

### 3.8. Statistical Analysis

Data were analyzed using SPSS version 17.0 (SPSS, Inc., Chicago, IL, USA) and expressed as mean ± standard error of mean (SEM). Three or more independent experiments were performed for the different assays. Results were expressed as percent inhibition of control (as 100%). Differences between groups were analyzed by student’s t-test or one-way analysis of variance (ANOVA) using Tukey’s *post-hoc* test and *p* values < 0.05 were considered significant.

## 4. Conclusion

We conclude that the antiplatelet and antithrombotic activities of tomato products and pomace were not modified by industrial tomato processing. Therefore, in terms of primary prevention of CVD, these results suggest that given the ability of pomace extract to inhibit platelet function, it could be considered as a lead source candidate as anti-platelet and antithrombotic agent.

## References

[1] World Health Organization (2004). WHO publishes definitive atlas on global heart disease and stroke epidemic. Indian J. Med. Sci..

[2] Laslett L., Alagona P., Clark B., Drozda J., Saldivar F., Wilson S., Poe C., Hart M. (2012). The worldwide environment of cardiovascular disease: prevalence, diagnosis, therapy, and policy issues: A report from the American College of Cardiology. J. Am. Coll. Cardiol..

[3] Fuentes E., Fuentes F., Andrés V., Pello O., de Mora J., Palomo I. (2013). Role of platelets as mediators that link inflammation and thrombosis in atherosclerosis. Platelets..

[4] Willoughby S., Holmes A., Loscalzo J. (2002). Platelets and cardiovascular disease. Eur. J. Cardiovasc. Nurs..

[5] Nishijima K., Kiryu J., Tsujikawa A., Miyamoto K., Honjo M., Tanihara H., Nonaka A., Yamashiro K., Katsuta H., Miyahara S. (2004). Platelets adhering to the vascular wall mediate postischemic leukocyte-endothelial cell interactions in retinal microcirculation. Investig. Ophthalmol. Vis. Sci..

[6] Jackson S.P., Nesbitt W.S., Westein E. (2009). Dynamics of platelet thrombus formation. J. Thromb. Haemost..

[7] Zoungas S., McGrath B.P., Branley P., Kerr P.G., Muske C., Wolfe R., Atkins R.C., Nicholls K., Fraenkel M., Hutchison B.G. (2006). Cardiovascular morbidity and mortality in the Atherosclerosis and Folic Acid Supplementation Trial (ASFAST) in chronic renal failure: A multicenter, randomized, controlled trial. J. Am. Coll. Cardiol..

[8] Hartley L., Igbinedion E., Holmes J., Flowers N., Thorogood M., Clarke A., Stranges S., Hooper L., Rees K. (2013). Increased consumption of fruit and vegetables for the primary prevention of cardiovascular diseases. Cochrane Database Syst. Rev..

[9] Plaza M., Cifuentes A., Ibáñez E. (2008). In the search of new functional food ingredients from algae. Trends Food Sci. Technol..

[10] Fuentes E., Astudillo L.A., Gutierrez M.I., Contreras S.O., Bustamante L.O., Rubio P.I., Moore-Carrasco R., Alarcon M.A., Fuentes J.A., Gonzalez D.E. (2012). Fractions of aqueous and methanolic extracts from tomato (Solanum lycopersicum L.) present platelet antiaggregant activity. Blood Coagul. Fibrinolysis.

[11] Fuentes E., Castro R., Astudillo L., Carrasco G., Alarcon M., Gutierrez M., Palomo I. (2012). Bioassay-guided isolation and HPLC determination of bioactive compound that relate to the antiplatelet activity (adhesion, secretion, and aggregation) from solanum lycopersicum. Evid.-Based Complement. Altern. Med..

[12] Fuentes E., Alarcon M., Astudillo L., Valenzuela C., Gutierrez M., Palomo I. (2013). Protective mechanisms of guanosine from solanum lycopersicum on agonist-induced platelet activation: Role of sCD40L. Molecules.

[13] Le Gall G., Colquhoun I.J., Davis A.L., Collins G.J., Verhoeyen M.E. (2003). Metabolite profiling of tomato (Lycopersicon esculentum) using 1H NMR spectroscopy as a tool to detect potential unintended effects following a genetic modification. J. Agric. Food Chem..

[14] Fuentes E., Carle R., Astudillo L., Guzmán L., Gutiérrez M., Carrasco G., Palomo I. (2013). Antioxidant and antiplatelet activities in extracts from green and fully ripe tomato fruits (solanum lycopersicum) and pomace from industrial tomato processing. Evid.-Based Complement. Altern. Med..

[15] Lavelli V., Torresani M.C. (2011). Modelling the stability of lycopene-rich by-products of tomato processing. Food Chem..

[16] Takeoka G.R., Dao L., Flessa S., Gillespie D.M., Jewell W.T., Huebner B., Bertow D., Ebeler S.E. (2001). Processing effects on lycopene content and antioxidant activity of tomatoes. J. Agric. Food Chem..

[17] Abushita A.A., Daood H.G., Biacs P.A. (2000). Change in carotenoids and antioxidant vitamins in tomato as a function of varietal and technological factors. J. Agric. Food Chem..

[18] Collins B., Hollidge C. (2003). Antithrombotic drug market. Nat. Rev. Drug Discov..

[19] Palomo I., Toro C., Alarcon M. (2008). The role of platelets in the pathophysiology of atherosclerosis (Review). Mol. Med. Rep..

[20] Hubbard G.P., Wolffram S., Lovegrove J.A., Gibbins J.M. (2004). Ingestion of quercetin inhibits platelet aggregation and essential components of the collagen-stimulated platelet activation pathway in humans. J. Thromb. Haemost..

[21] Hu F.B. (2003). Plant-based foods and prevention of cardiovascular disease: An overview. Am. J. Clin. Nutr..

[22] Capanoglu E., Beekwilder J., Boyacioglu D., Hall R., de Vos R. (2008). Changes in antioxidant and metabolite profiles during production of tomato paste. J. Agric. Food Chem..

[23] Schieber A., Stintzing F.C., Carle R. (2001). By-products of plant food processing as a source of functional compounds-recent developments. Trends Food Sci. Technol..

[24] Schweiggert R.M., Mezger D., Schimpf F., Steingass C.B., Carle R. (2012). Influence of chromoplast morphology on carotenoid bioaccessibility of carrot, mango, papaya, and tomato. Food Chem..

[25] Guyatt G.H., Akl E.A., Crowther M., Gutterman D.D., Schuunemann H.J. (2012). Executive summary: Antithrombotic therapy and prevention of thrombosis, 9th ed: American college of chest physicians evidence-based clinical practice guidelines. Chest.

[26] Born G.V., Cross M.J. (1963). The aggregation of blood platelets. J. Physiol..

[27] Przyklenk K., Whittaker P. (2007). Adaptation of a photochemical method to initiate recurrent platelet-mediated thrombosis in small animals. Lasers Med. Sci..

